# Association of *Mycobacterium avium* subsp. *paratuberculosis* with Multiple Sclerosis in Sardinian Patients

**DOI:** 10.1371/journal.pone.0018482

**Published:** 2011-04-13

**Authors:** Davide Cossu, Eleonora Cocco, Daniela Paccagnini, Speranza Masala, Niyaz Ahmed, Jessica Frau, Maria Giovanna Marrosu, Leonardo A. Sechi

**Affiliations:** 1 Dipartimento di Scienze Biomediche, Sezione di Microbiologia e Virologia, Università di Sassari, Sassari, Italy; 2 Centro Sclerosi Multipla, Dipartimento di Scienze Cardiovascolari e Neurologiche, Università di Cagliari, Cagliari, Italy; 3 Pathogen Biology Laboratory, Department of Biotechnology, School of Life Sciences, University of Hyderabad, Hyderabad, India; 4 Institute of Biological Sciences, University of Malaya, Kuala Lumpur, Malaysia; National Institute for Infectious Diseases (L. Spallanzani), Italy

## Abstract

*Mycobacterium avium* subsp. *paratuberculosis* (MAP) infection is highly spread in the ruminant herds of Sardinia, in the Western Mediterranean. The objective of this study was to investigate prevalence of MAP infection in association with Multiple Sclerosis (MS) using clinical specimen from patients and controls. We analyzed samples for the presence of MAP specific DNA and to demonstrate humoral response to a MAP protein (MAP2694), a predicted homologue of the T-cell receptor gamma-chain/complement component 1 of the host. We found presence of MAP DNA in 42% of the MS patients and an extremely significant humoral immune response revealed by the MS patients against the MAP protein. In our opinion, this is the first report that significantly associates MAP infection with MS. Further studies will be required to confirm if MAP could be one of the triggers of MS, according to the molecular mimicry theory, in susceptible (and genetically at risk) individuals.

## Introduction

Multiple sclerosis (MS) is a chronic, autoimmune and demyelinating syndrome that primarily affects the central nervous system. MS is characterized by the infiltration of myelin-epitope–specific CD4+ T cells [1] that attack constituents of the axonal myelin sheath and other elements of the central nervous system (CNS), destroying myelin and the basal axon. Moreover, γδ T cells play a role in the development and potentially the progress of the demyelinating CNS inflammation [2].

The etiology of MS is largely elusive, although it is commonly believed to be caused by the joint action of several, largely unknown, genetic factors and susceptibility variants in the presence of other unidentified, permissive/triggering environmental agents. Among the non-heritable causative factors hypothesized to be involved in MS etiology and pathogenesis is MAP, a potentially infective, obligate pathogenic bacillus of the genus Mycobacterium that causes Johne's disease in ruminants and it is associated to a human form of the enteropathy called Crohn's disease (CD) [3]; more recently, it was linked to type 1 diabetes mellitus (T1DM) [4]. Polymorphisms in the natural resistance-associated macrophage protein gene (*SLC11A1*, ex *NRAMP1*) have previously been found to be associated with mycobacterial infections as well as CD, T1DM and MS [4], [5], [6]. NRAMP1 is an iron transporter associated with macrophage activation. This gene has multiple pleiotropic effects on macrophage function, including regulation of cytokine production, tumor necrosis factor α, interleukin-1 β, inducible nitric oxide synthase and regulation of major histocompatibility complex class II expression and antigen presentation functions [7], [8]. All of these activities are not only essential for protection against mycobacterial infection (innate defenses), but also critically involved in the induction and progression of autoimmune diseases [5], [7], [8].

MS distribution in Europe follows a north-south gradient, with the notable exception of the Mediterranean island of Sardinia, which has one of the highest prevalence worldwide together with a reportedly high incidence of T1DM [5], [9]. These data suggest that T1DM and MS might be triggered by a common environmental/infectious agent in Sardinia and that its population could be a suitable target to explore because of their genetic peculiarity which might have been shaped over time as a function of strict isolation and consequent endogamy, consanguinity and the burden of historical scourges such as Malaria. High resolution, population based analyses are required to ascertain or negate any role of the unique Sardinian haplotypes and the high incidence of the autoimmune diseases.

In view of the above and since molecular mimicry between human and MAP proteins was postulated as a mechanism for Crohn's disease and T1DM [4], [9], [10], we wanted to look for the presence of MAP in MS patients by PCR and/or immunoassays against MAP2694, which is a transmembrane glycine and proline rich protein of 351 amino acids conserved in the Mycobacteriaceae family.

## Materials and Methods

### Subjects

A total of 50 MS patients [28 females & 22 males (F/M:1.2/1); 42 relapsing-remitting, 6 secondary progressive, 2 primary progressive - actual age = 44.5 (SD+/−11.25), age at onset = 30.4 (SD+/−9.9), disease duration from onset = 14.1 (SD+/−11.6)] and 56 ethnically and demographically similar healthy controls [30 females & 26 males (F/M: 1.1), actual age = 47.7 (SD+/−12.7), including people related to patients such as friends and spouses but not direct relatives] were enrolled in the study. No statistically significant differences with respect to gender, age and descent were observed among patients and healthy groups. All the patients had clinically definite MS according to the McDonald Criteria (version 2005) [11] and were free from any immunomodulating or immunosuppressive drugs for at least 6 months.

### Ethical statement

Blood samples were collected after obtaining informed written consents from all subjects. The study protocols were approved by the ethics committee of the University of Cagliari, Sardinia, Italy.

### IS900 MAP PCR

The presence of MAP specific DNA was ascertained by observing IS900 specific amplicons after PCR was performed as described previously [3].

### MAP 2694 Cloning and Expression

Specific primers were designed to amplify MAP2694 from the genome of MAP strain ATCC43015. The amplified fragment of 1056 bp was inserted into the pMAL-c2x vector between *BamH1* and *Pst1* cleavage sites. The ligation mixtures were used to transform *E. coli* K12 TB1cells. After induction with IPTG, the expressed protein was purified with three column purification steps for each extraction. A specific fraction corresponding to our recombinant/fusion protein(s), at the level of 78.5 kDa, was observed on the SDS-PAGE gel. This was in agreement with the expected collective molecular mass of the fusion proteins [maltose binding protein (MBP) (42.5 kDa) and MAP2694 (36 kDa]. Western blotting confirmed that the protein was recognized by human sera (that were tested positive to previously expressed MAP proteins) indicating that the purified protein could be used as an antigen for immuno-diagnostic tests (see results).

### ELISA

The immunological screening was performed by ELISA wherein all assays were performed in replicates as previously reported [4] and the results were expressed as means ± standard error of the mean. For the sake of negative control, ELISA assays using the *E. coli* extracts containing the vector without the insert and expressing only the MBP were performed. No reaction was observed [optical density (OD) values were under 0.2 in both MS patients and controls]. All values were checked for significance by the Chi-Square test with Yate's corrections. ELISA performance was also assessed by the area under the receiver operating characteristic curve (AUC-ROC).

## Results

A total of 21 out of 50 MS patients were observed positive by PCR (42%) based on the amplification of IS900, a specific signature element within the genome of MAP, whereas, only 7 out of 56 samples in the control group were observed positive (12.5%) (Table 1). Statistical analysis by Chi-square test generated a value of 10.687 with 1 degree of freedom. The two-tailed *P* value was equal to 0.0008; the association was thus found very significant.

**Table 1:** ELISA and MAP-PCR results.

	MAP2694 antibody- positive	MAP2694 antibody-negative	IS900PCR +	IS900PCR −
MS patients (n = 50)	16(32%)	34(68%)	21(42%)	29(58%)
Healthy controls (n = 56)	1(2%)	55(98%)	7(12%)	49(87%)
Total	17	89	28	78

Antibody positive patients were so identified by selecting a cut-off OD value of 0.84 (as calculated by the mean plus two times the standard deviation of the mean).

**Figure 1:**
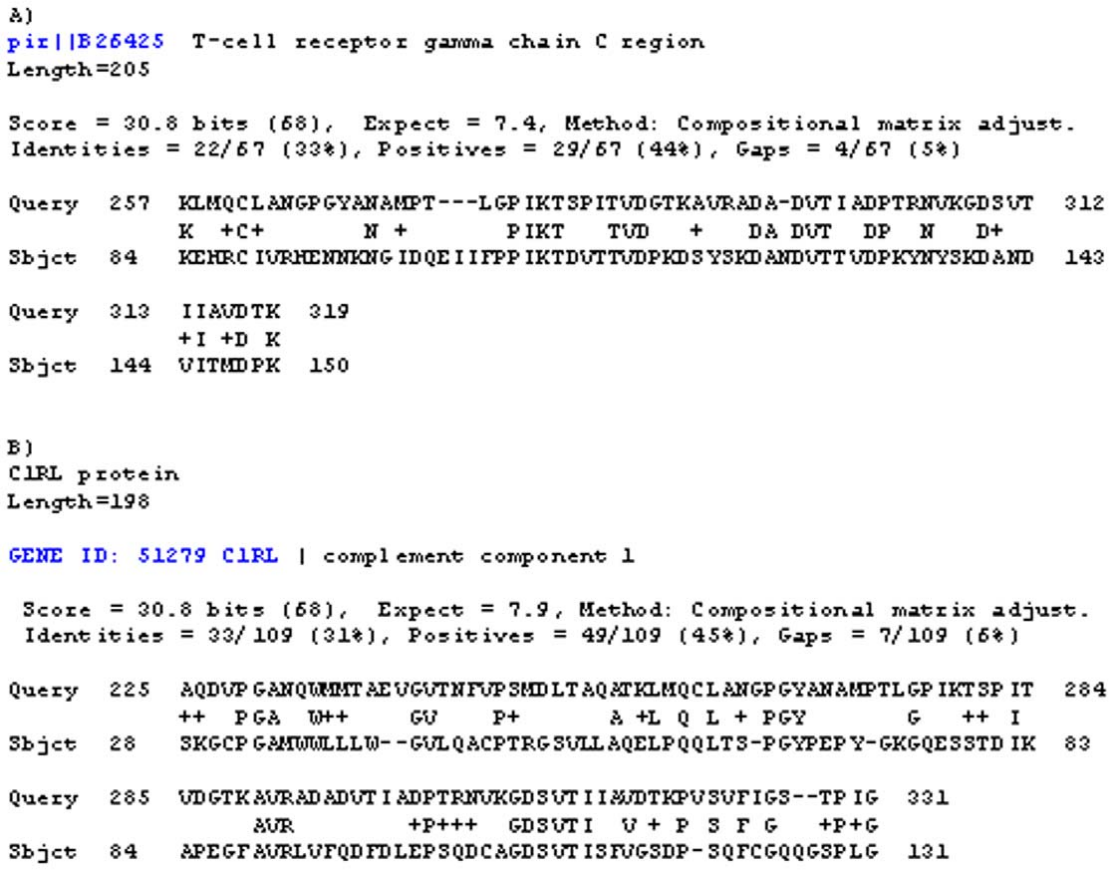
Blast alignments with masking-off segments of the query sequence that have low compositional complexity. Results show region of identity, strongly similar amino acids with a plus (+) and missing region in dashes (–). Query sequence is MAP2694.

**Figure 2:**
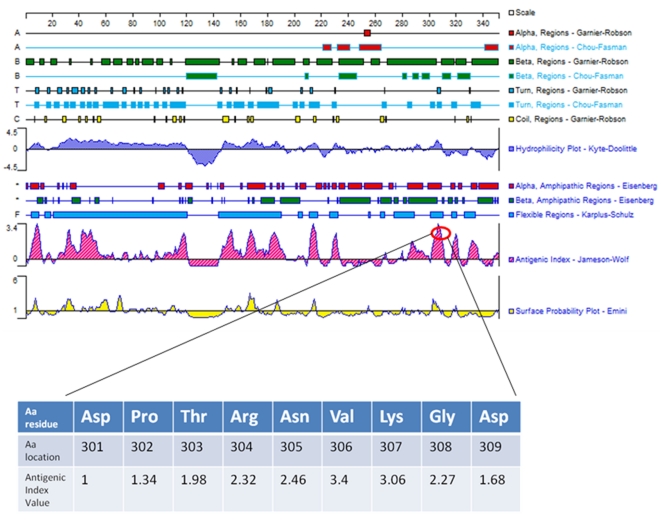
Computational prediction of the MAP2694 structure and properties such as different helices and turns, hydrophobicity, antigenicity and surface probability, etc. using Protean software from the DNAstar package (DNAstar Inc., Madison, USA). In the inset is the highlighted antigenic index value which corresponded to the homology between MAP2964 (Aa 301-209) and T-cell receptor gamma/complement component 1.

A MAP specific gene was identified by comparisons between the MAP genome [12] and the repertoire of human genes by using NCBI/BLAST. After BLAST -P analysis, we identified MAP2694 to share sequence homology to the T-cell receptor gamma chain C region (Figure 1A, Aa region 257–319) and also to the complement component 1 (Figure 1B, Aa region 225–331). Analysis of predicted antigenic attributes of MAP2964 by the DNAStar program is shown in Figure 2. The values indicating antigenic index, hydrophilicity and surface probability are are seen therein. A possibly significant epitope (max antigenic index 3.4) was identified and highlighted on the image between amino acids 301–309. It is located within the homology between MAP2964 and T cell receptor gamma chain/complement component 1 of the host and it has a high probability to be exposed on the surface of the membrane (max 2.39).

In view of these putative homologies, it is noteworthy that the γδ T cells have the potential to influence all levels of inflammation through rapid production of inflammatory mediators, recruitment of inflammatory cells *via* chemokines, influencing T cell differentiation by cytokine production, and/or direct killing *via* production of cytotoxic mediators [2], [13]. Many data suggest that γδ T cells play a pathogenic role in CNS inflammation and autoimmunity [2], [13].

Complement activation is known to occur in (white matter) MS lesions. It is thought to mediate oligodendrocyte/myelin damage and to be a marker of pathologic heterogeneity among individuals [14]. Deposition of the complement activation product, C1q was detected on and within macrophages/microglia and astrocytes and in blood vessel walls in (white matter) MS lesions [14].

**Figure 3:**
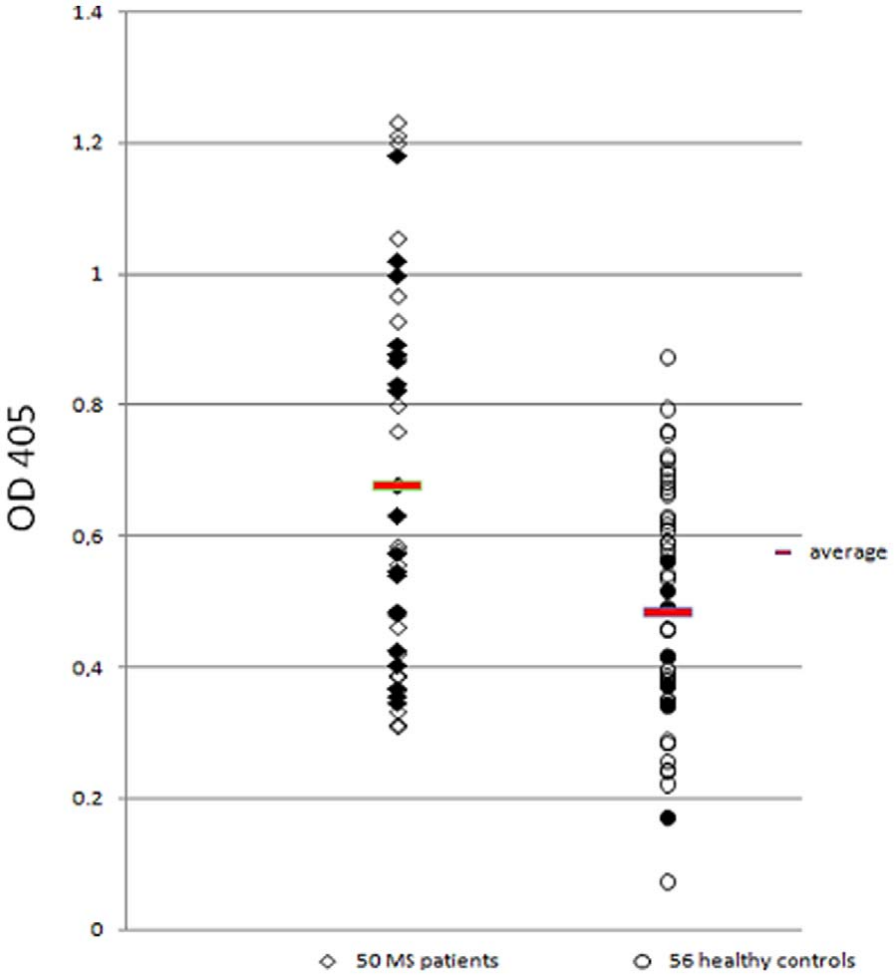
Evaluation of serum samples against MAP2694 recombinant protein. MS patients sera are shown in the left column whereas Healthy Controls sera are shown in the right column. Data are presented as OD values observed in ELISA, as also described in the text. Data from a representative experiment out of three are shown. The mean value for each group is indicated by a thick horizontal line. IS900 PCR positive samples are indicated as black solid figures.

**Figure 4:**
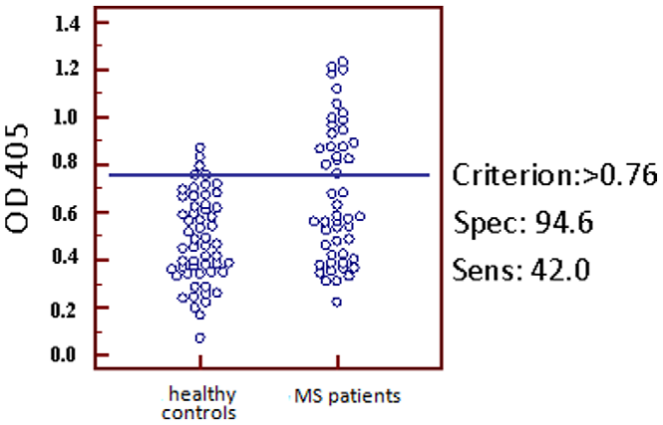
AUC-ROC Test. Evaluation of serum samples from 50 MS patients (right column) and 56 healthy controls (left column) against MAP2694 recombinant protein by the AUC-ROC test. The horizontal blue line highlights the cut-off value(s) which gives a specificity of 25%. Data are presented as OD values observed in ELISA. Area under curve (AUC) = 0,686; P = 0,0003.

These observations led us further to design immunoassays for the detection of anti-MAP antibodies in this group of patients. The MAP2694 antigen gave strong ELISA values in 32% of the sera of MS patients and only in 2% of the controls (chi = 16.04; P<0.0001) (Figure 3 and Table 1). Such extremely significant humoral responses to the recombinant MAP2694 corroborated with and strengthened our initial hypothesis. The performance of ELISA test was also assessed by the area under the receiver operating characteristic curve (AUC-ROC) (Figure 4) where the best cut off value is indicated at 0.784 with a sensitivity of 42% and an extremely statistically significant P value of 0.0003. It is interesting to observe that both the patients with primary progressive disease were simultaneously positive to PCR as well as ELISA whereas, among the 6 patients with secondary progressive disease, one was positive to both PCR and ELISA, three were positive only to PCR, and 2 were negative to both the tests.

## Discussion

Several studies have suggested that different pathogens could trigger an autoimmune response by immunological cross-reactivity or molecular mimicry [15]. Sardinia acts as an ideal setting for MS studies because the prevalence of MS in the region is perhaps the highest observed in the world. Epidemiological evidences support the idea that the gene pool of Sardinians has been subjected to selective pressures during the history, but dramatically high incidence of MS in Sardinia could not be explained only by genetic predisposition; this necessitates search for a strong environmental trigger also.

Our finding of MAP PCR positivity in 42% of the MS patients and its corroboration with the humoral responses observed against MAP2694 protein, homologue of a γ T-cell receptor and the complement component 1 of the host, appears to be a potential start point to gain more in depth mechanistic insights in to the development of MS and other autoimmune diseases which are supposedly under the regulatory control of an environmental trigger to induce autoimmunity. However, not all of our healthy controls were negative for the presence of MAP. But, as previously found [3], [4], [6], [9], [10], a low percentage of healthy controls gave a positive test for MAP pointing to the possibility that the patient population might have been exposed to MAP and depending on individual genetic susceptibility, the infection may be contained or may have progressed to a chronic autoimmune stage. In contrast with the previous findings [16], we found a poor correlation between PCR results and serological response to MAP antigens. We suggest that ELISA positivity may follow different patterns than PCR positivity due to the immunological statuses of the patients which might possibly be anergic or have an altered immune response. It is interesting to observe that PCR was positive in the two cases of primary progressive disease and in 4 out of 6 cases of secondary progressive disease.

Homology of the MAP2694 to the T-cell receptor γ chain and the complement component 1 is an important observation which is potentially suggestive of an underlying molecular mimicry.

γδ T cells are a family of cells which have a role in both innate and adaptive immunity [2], [13]. The fact that they are commonly known for their response to mycobacterium and their locations at mucosal sites is very intriguing in light of our results. γδ T cells have been located in the CSF and in lesions of MS patients and clearly they seem to have a role in the regulation of autoimmune inflammation in the CNS and in other similar disorders such as diabetes and arthritis [17], [18].

Kobayashi *et al* [19] administered T-cell receptor (TCR) γδ -specific monoclonal antibody (mAb),UC7-13D5, to elucidate the potential role of γδ T-cells in the pathogenesis of experimental, allergic encephalomyelitis (EAE) in mice. mAb treatment led to transient depletion of the γδ– T cells *in vivo*. mAb-treated EAE mice showed an acceleration of the disease onset, as well as relapse, and, also increased Ag-specific proliferative responses compared to control mice implying a protective function of T cells [18]. This may be suggestive of a mechanism where antibodies raised against MAP2964 may cross react as depleting antibodies against γδ T-cells which in turn accelerate the disease. This has led us to speculate that MAP infection (or other mycobacteria) could be central to the pathogenesis of MS. The fact that we have previously found relevant *NRAMP1* polymorphisms in Crohn's patients [6], T1DM patients [4] and MS patients [5], together with the positive results given by IS900 PCR, suggests that there is a strong possibility of MAP being involved with autoimmune responses in MS just in the same manner as it did in Crohn's and T1DM.

In conclusion, these first and potentially relevant observations reinforce our preliminary hypothesis that MAP could be one of the triggers of MS (conforming to the molecular mimicry theory) in susceptible (and genetically at risk) individuals [15]. This finding might stimulate further studies to verify our pilot observations and open new frontiers for the investigation of pathogenesis of MS, including the possibility to develop new intervention strategies.
